# Effect of low-dose dexketoprofen trometamol and paracetamol on postoperative complications after impacted third molar surgery on healthy volunteers: A pilot study

**DOI:** 10.4317/medoral.19835

**Published:** 2014-08-17

**Authors:** Cennet N. Eroglu, Ercan Durmus, Demet Kiresi

**Affiliations:** 1 D.D.S., PhD., Assist. Prof., Department of Oral and Maxillofacial Surgery, Yuzuncu Yil University Faculty of Dentistry, Van, Turkey; 2 D.D.S., PhD., Prof., Department of Oral and Maxillofacial Surgery, Selcuk University Faculty of Dentistry, Konya, Turkey; 3 M.D., Assoc. Prof., Department of Radiology, Konya Necmettin Erbakan University Meram Faculty of Medicine, Konya, Turkey

## Abstract

Objectives: The aim of the present study was to investigate the analgesic and anti-inflammatory effects of dexketoprofen trometamol (DT) and paracetamol on deep acute somatic pain and inflammation in patients undergoing impacted third molar surgery. This study was planned to present benefits that we could obtain with low burden of drug. Study Design: Effects of drugs, which were administered preemptively before the procedure, on pain, mouth-opening limitation, and swelling were assessed by visual analogue scale (VAS), magnetic resonance imaging (MRI), and mouth-opening measurement. Following surgery, time intervals when the patients first need to receive the drug were measured. Results: The VAS scores of the patients were lower in the side treated with DT than that in the paracetamol treated side. There was no significant difference between the groups in terms of mouth-opening limitation. MRI recordings revealed that swelling was lower in the side treated with paracetamol than DT treated side. Conclusions: Administration of the drugs before surgery contributed to the postoperative patient comfort. The analgesic activity of 12.5 mg dose of DT was similar to, even better than, the analgesic activity of 500 mg dose of paracetamol; however, DT had insufficient anti-inflammatory efficacy.

** Key words:**Dexketoprofen trometamol, paracetamol, magnetic resonance imaging.

## Introduction

Dexketoprofen trometamol (DT), which has been in clinical use since 1996, is the water-soluble salt of dextrorotatory enantiomer of racemic ketoprofen, a non-steroidal anti-inflammatory drug (NSAID) (1). Since dexketoprofen is more lipophilic than ketoprofen, it is rapidly absorbed, its activity starts in a short time and it reaches to maximum plasma concentration in a short period (2,3). Dexketoprofen presents its peripheral activity directly on the lesion area and central activity directly on the central nervous system (4,5). Likewise other NSAIDs, antipyretic, anti-inflammatory, and analgesic mechanisms of action of dexketoprofen are based on the inhibition of prostaglandin synthesis.

Paracetamol is a non-opioid analgesic preferred in mild-to-moderate pain due to its weak inhibition effect on prostaglandin synthesis, although its mechanism of action is not clearly defined. Despite its short half-life, in addition to its solitary usage as an analgesic, paracetamol is used to support NSAIDs or as an alternative option where NSAIDs are contraindicated in history of hypersensitivity, bleeding and gastrointestinal ulcer (6). Its anti-inflammatory property is rarely mentioned while listing all these properties.

The present study including healthy volunteers with bilateral impacted mandibular third molars investigated the efficacy of drugs, which belong to two different drug classes (propionic acid class of NSAIDs and para-aminophenol derivative, non-opioid), during the inflammatory process expected to occur following the extraction of impacted wisdom tooth in the same individual. The right or left impacted wisdom teeth of the patients were randomly allocated into DT and paracetamol groups. DT and paracetamol were commenced before the operation at doses of 12.5 mg and 500 mg, respectively, those do not cause adverse effects, and continued after the operation in order to evaluate whether they were effective on acute deep somatic pain, swelling, and trismus.

## Material and Methods

In the present prospective study, 21 outpatients between 18 and 35 years of age who had bilateral impacted lower third molars requiring removal of alveolar bone for extraction and were admitted to the Department of Oral and Maxillofacial Surgery of Selcuk University Faculty of Dentistry were enrolled. The study protocol was approved by the Research and Ethics Committee of the Faculty of Dentistry and the informed consents were obtained.

The study population was composed of healthy volunteers having no systemic disease or available for MRI evaluation. In clinical and radiological examinations, it was paid attention that right and left mandibular wisdom tooth symmetrically be in the same angulation and impacted with the same depth. The right or left impacted wisdom teeth of the patients were randomly allocated into DT and paracetamol groups; there was no placebo group. In the early preoperative period, the patients received a single initial dose of either 12.5 mg DT or 500 mg paracetamol one hour before the operation. Each patient received both of the treatments in random order and they were operated by the same surgeon. In all surgical interventions, mucoperiosteal flap and osteotomy were performed. For maintenance of anesthesia, 4% articaine HCl with 1:100,000 epinephrine was used. Surgery was performed twice for each patient, at first, one impacted tooth was removed and two weeks later, in case no symptoms arose from the first surgical procedure, other impacted tooth was removed.

After the operation, all patients were administered 625 mg dose of amoxicillin/clavulanic acid two times a day for five days and chlorhexidine mouthwash two times a day for seven days. We prescribed prophylactic antibiotics for our patients against the risk of infection. In case of postoperative pain, patients were asked to receive the preoperative doses of the same drugs (500 mg paracetamol or 12.5 mg DT) for every 8 hours. Each patient was informed about how to measure pain intensity on a VAS ranging from 0 to 10; 0 represented no pain and 10 represented the worst pain imaginable. The patients were asked to receive the same drug whenever moderate or severe pain (score ≥4 on VAS) arose after the surgery and to record the dosing time and VAS score at that time on the recording form for 24 hours. Study investigator collected the forms on the 2nd postoperative day after the 1st postoperative clinical control was performed.

Maximum mouth opening was measured by a vernier-calibrated sliding caliper preoperatively and on the 2nd and 7th postoperative days in order to assess trismus. Trismus on the 2nd and 7th postoperative days was assessed based on the following calculations: 1) preoperative maximum mouth opening - 2nd postoperative day maximum mouth opening = 2nd postoperative day trismus value, 2) preoperative maximum mouth opening - 7th postoperative day maximum mouth opening = 7th postoperative day trismus value.

Swelling was evaluated by magnetic resonance imaging (MRI). All examinations were performed by the same radiologist in the Department of Radiology of Konya Necmettin Erbakan University Meram Faculty of Medicine. Each patient was evaluated in the preoperative period and on the 2nd postoperative day. MRI records were obtained at a workstation program using 1.5 Tesla, Picker PQS (Picker Edge, Cleveland, Ohio, USA) with a head coil. Images were obtained by an axial spin-echo T1-weighted sequence with 5-mm slice thickness. Slices were taken parallel to the occlusal plane and perpendicular to the mandibular alveolar process. The distance between the mandibular alveolar process-skin was measured.

Data were analyzed using the statistical package for the social sciences (SPSS Inc., Chicago, IL, USA) version 7.5. The independent Student’s t-test was used to compare data between the two study drug groups. A p value <0.05 was considered statistically significant.

## Results

In the present study, 6 out of 21 outpatients were excluded from the study, of whom 2 did not undergo the second scheduled surgery, 2 had insufficient MRI results, and 2 did not comply with the drug regimen. Thus, the remaining 15 patients were included in the analysis. The mean age of the patients was 22.8±1.41 years and the male-to-female ratio was 1:2. All the values and measurements (VAS scores, swelling, and trismus values) for each patient are presented in  Table 1.

**Table 1:**
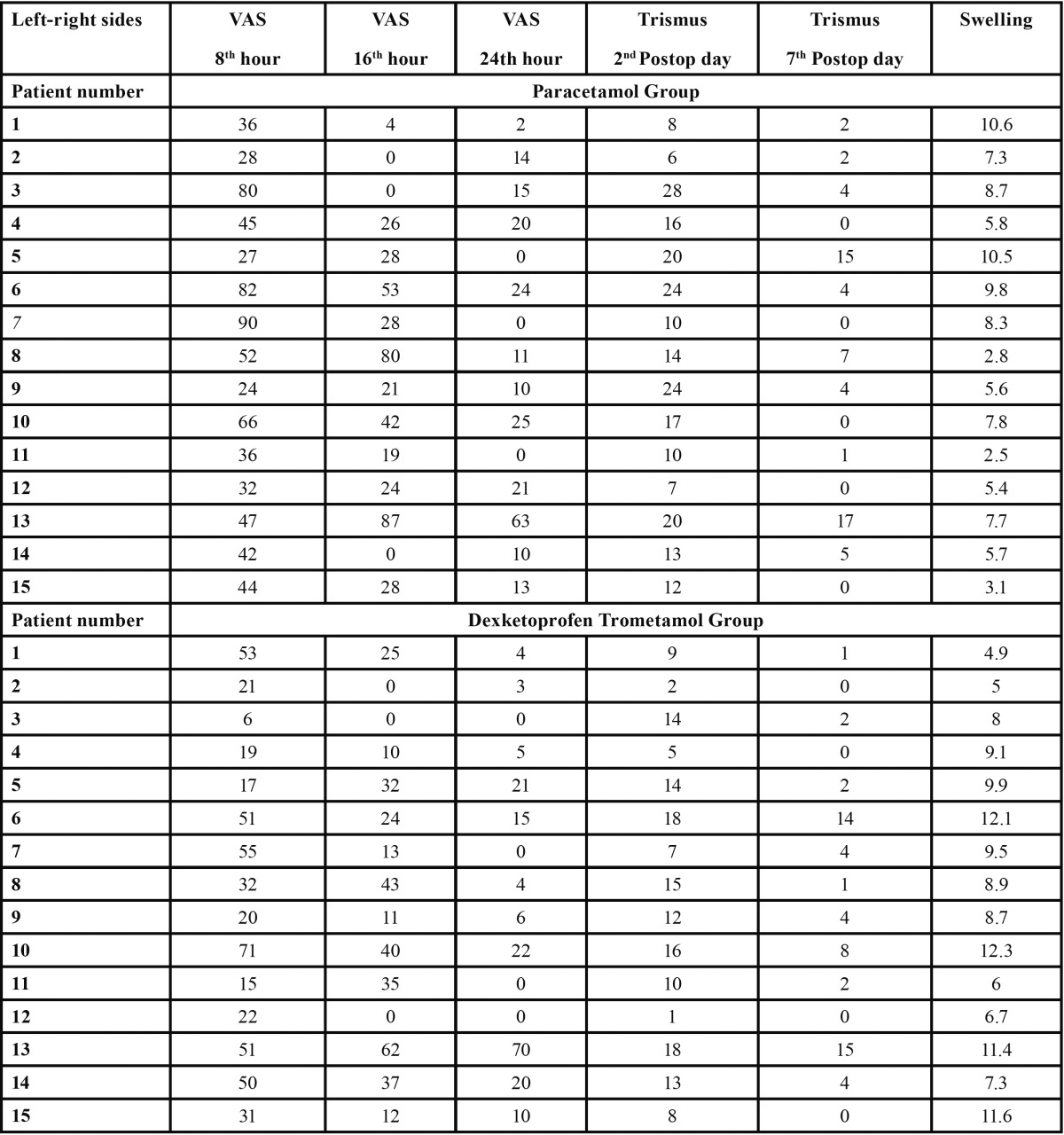
All values and measurements (VAS scores, swelling, and trismus values) for each patient.

Time to the first need for drug after the surgery were increased in both groups and the most noted time interval on the forms was the 5th and 6th hour interval after the first use of drug. Only 1 patient noted the 3rd and 4th hour interval.

There were no significant differences between the two groups in terms of the postoperative VAS scores of 8th, 16th and 24th hours (*p*=0.006, *p*=0.452, *p*=0.607, respectively). However, in the DT group, analgesia was more effective at a rate of 33%, 22%, and 21% on the 8th, 16th, and 24th hours, respectively as compared to those in the paracetamol group.

In the evaluation of swelling between the groups in terms of preoperative and postoperative measurements, the values in the DT group were significantly higher on the 2nd postoperative day than that in the paracetamol group. The paracetamol group demonstrated a 22% lower rate of swelling than the DT group.

The patients in the DT group had less trismus values than those in the paracetamol group on the 2nd and 7th postoperative days, although statistically not significant (*p*=0.56 and *p*=0.887, respectively). While the patients in the paracetamol group presented better trismus values than DT group by 29% on the 2nd postoperative day, the difference was only 7% on the 7th postoperative day. In both drug groups, the mean trismus values of males were significantly lower than those of the females (*p*=0.001 for both). The study results are summarized in  Table 2.

**Table 2:**
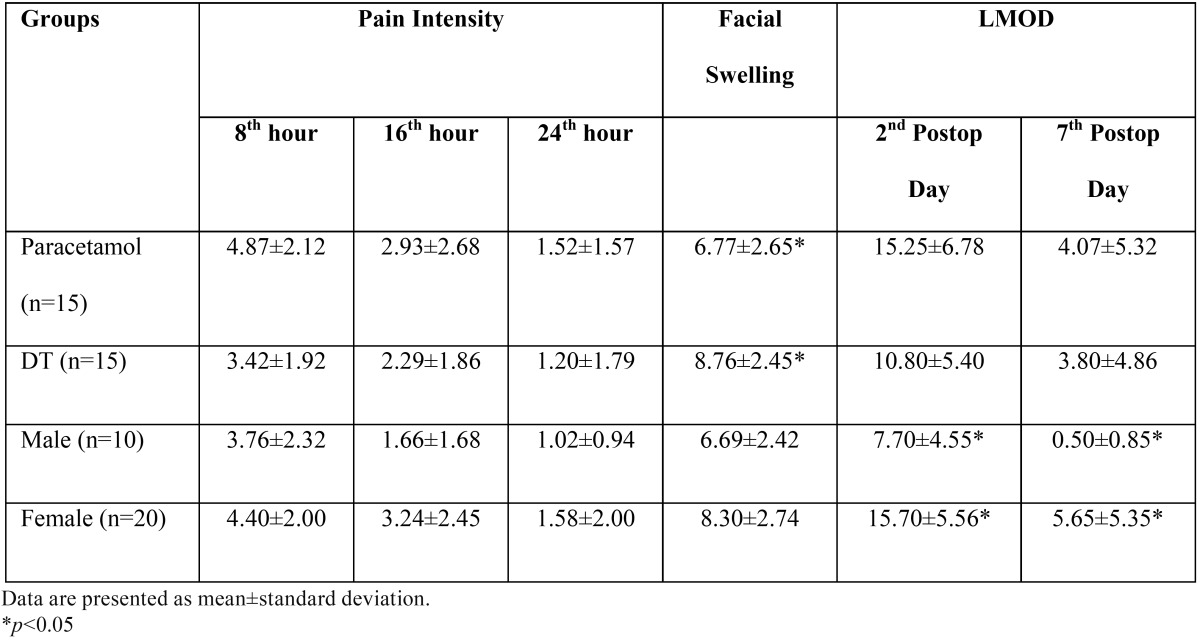
Pain intensity, facial swelling, and limited mouth opening difference measurements of patients according to the study groups and gender.

## Discussion

Severity of the inflammatory response may vary even the reason for local tissue damage in the extraction of impacted wisdom tooth is similar for every patient (7). It has been suggested that administering the drug before procedure alleviates the inflammatory process and reduces the sequelae of tissue trauma and particularly pain (8). With regard to the suppression of inflammatory process in particular, presence of drug in the metabolism during the procedure would decrease the number of mediators likely to be released from the tissue in the surgical field.

Researches concerning bone surgery have proven that DT, which is considered to be a good pain reliever, reinforces anesthetic block and reduces need for postoperative analgesic and morphine when administered before the procedure (9). In the present study as well, administration of analgesic before the procedure prolonged the post-procedure pain-free period and the postoperative initial doses of both analgesics were received later than expected. When we considered the mean duration of local anesthesia as 3-3.5 hours based on the anesthetic agent used, time of the earliest drug use was recorded between the postoperative 5th and 6th hours. A study comparing the postoperative single doses of NSAIDs reported analgesic efficacy similar to that in the present study but with 25 mg and 50 mg doses of DT (10).

In non-surgical pain such as dental pain, dysmenorrhea, headache, and pain of musculoskeletal disorders, DT has been emphasized that it is safe in oral treatments and may be the first choice for therapeutic indications among 9 different drugs when compared with paracetamol-metamizole control groups (11). However, in addition to overall prospectus information and research results, the most important thing to be considered about DT is the fact that patients with hepatic impairment can tolerate DT at a single daily dose of 25 mg, whereas patients with mild-to-moderate renal insufficiency can tolerate DT at a single daily dose of 12.5 mg (12,13). For this reason, the outcomes of 12.5 mg DT were investigated in the present study.

In postoperative dental pain, comparison of 12.5 mg and 25 mg doses of DT with 575 mg dose of dipyrone revealed that DT had more analgesic efficacy than dipyrone even at a dose of 12.5 mg (14). In the present study, 12.5 mg of DT provided a better analgesic effect than 500 mg of paracetamol according to the VAS scores of the patients, particularly in the first 8th hour after the surgery in which the pain level was expected to be the highest. Studies have mostly stressed on the pain reliever property of DT rather than its anti-inflammatory property. Only a single study which was performed following the extraction of impacted wisdom teeth compared the anti-inflammatory activity of DT with that of another NSAID; however, the most remarkable improvable point of this study was that the evaluation of inflammation was not presented according to an objective criterion (15). In the present study, determination of the amount of swelling by MRI and measurement of mouth-opening limitation provided more objective data to evaluate anti-inflammatory effect of DT. In addition to any side effects of NSAIDs, photo-contact dermatitis, neutropenia, thrombocytopenia, liver injury (after 10 days use), acute renal injury, and rhabdomyolysis (a single dose of 25 mg received for cold) have been reported in the literature as side the effects of DT (16-18). In the present study, higher amount of bleeding on the DT side as compared to the paracetamol side was observed during and after the surgery. However, further studies comparing DT with another NSAID in the same patients may provide more accurate results on whether DT enhances the bleeding.

In the present study, DT administered at low doses to avoid side effects was compared with paracetamol in terms of clinical out-comes. Better results were obtained in the VAS scores, in the number analgesics received postoperatively and in the pain-free interval by 12.5 mg dose of DT than 500 mg dose of paracetamol. However, 12.5 mg dose of DT was found to be ineffective on swelling as compared to paracetamol, while there was no significant difference between the groups in terms of trismus. There are also studies in which anti-inflammatory efficacy of paracetamol has been clinically compared and found to be better than, or equal to, that of NSAIDs. It has been reported that indomethacin, a NSAID, and acetaminophen/codeine have equally reduced the complications such as pain and swelling developed after the impacted third molar surgery (19). In another study, naproxen 500 mg b.i.d. and acetaminophen 1000 mg q.i.d. were administered to the patients after the third molar surgery, and swelling was observed comparatively less by 22.4% with acetaminophen on the postoperative 3rd day (20). In the other study from the same authors, paracetamol 1000 mg q.i.d. and ibuprofen 600 mg q.i.d. presented no significant difference in swelling after three days of administration; however, paracetamol was found to be more successful by 1.8% on the postoperative 3rd day (21). However, since anti-inflammatory efficacy of paracetamol is quite low, it is not considered among NSAIDs. Under the light of these information, the question to be asked is whether paracetamol is a more potent anti-inflammatory drug than some NSAIDs or this group of drugs has a mechanism that could lead to swelling. Considering that the classification of NSAIDs have been performed based on the likely response to an irritating substance on animals, to what extend the NSAIDs present efficacy in human body can be exposed by only such type of studies (20).

The present study revealed that the values of female patients were higher than those of male patients. While some of these values were statistically significant, some were only clinically significant. Although it has been defended that gender has an effect on swelling (22), it would be better to determine its role with further studies that would be performed in higher number of individuals.

Since pain is an expected process after extraction of impacted wisdom tooth, the present study did not comprise a placebo group. We assessed paracetamol, which was frequently used in clinical practice and considered both safe and sufficient for postoperative pain, as the control group. Each patient received both of the drugs to avoid personal differences in response. Surgeries were performed rapidly by a single experienced surgeon. Patients were monitored for the first 24 and 48 hours during which the pain and swelling were at the maximum level. Results were evaluated over objective criteria. In conclusion, the outcome of the present study revealed that DT, which was preemptively administered before surgery at safety dose limits according to the literature, had weak anti-inflammatory but adequate analgesic effects; however, paracetamol was both safe and satisfactory with regard to clinical outcomes.
